# Effects of Synergistic Massage and Physical Exercise on the Expression of Angiogenic Markers in Rat Tendons

**DOI:** 10.1155/2014/878095

**Published:** 2014-05-12

**Authors:** Waldemar Andrzejewski, Krzysztof Kassolik, Piotr Dziegiel, Bartosz Pula, Katarzyna Ratajczak-Wielgomas, Karolina Jablonska, Donata Kurpas, Tomasz Halski, Marzena Podhorska-Okolow

**Affiliations:** ^1^Department of Physiotherapy, University School of Physical Education, 35/p-4 Paderewskiego Street, 51-612 Wroclaw, Poland; ^2^Public Higher Medical Professional School, 68 Katowicka Street, 45-060 Opole, Poland; ^3^Department of Histology and Embryology, Wroclaw Medical University, 6a Chalubinskiego Street, 50-368 Wroclaw, Poland; ^4^Family Medicine Department, Wroclaw Medical University, 1 Syrokomli Street, 51-141 Wroclaw, Poland

## Abstract

Physical exercise and massage are regarded as key factors in regulating tendon structure. However, information on the mechanism through which massage influences the structure and biology of a tendon is scarce. In this study, we attempted to define the impact of these two activities on rat tendons by using morphological and molecular techniques, determining the expression of VEGF-A, FGF-2, and CD34 in the tendons of rats subjected to 10 weeks of physical exercise (running) with massage of varied duration. The group of rats that was trained and massaged during the entire study was characterized by the highest expression of these markers, compared to the rats subjected to massage before training and to the control group subjected to physical exercises only. The greatest significant differences, compared to the control, were noted in the expression of all the studied markers at mRNA level, and in the case of VEGF-A, at protein level, in the third and fifth weeks of the experiment. The results of this study could point to the synergistic impact of simultaneous massage and physical exercise on the expression of angiogenesis markers in rat tendons.

## 1. Introduction

Tendons are responsible for transferring the forces exerted by muscles to the bone tissue, thus enabling locomotion. It has been shown that tendons change their metabolism in response to mechanical forces and that, depending on the intensity of the mechanical stimulus, tendon fibroblasts secrete various proteins, such as collagen I and cyclooxygenase [1–5]. Although tendons are regarded as low metabolism tissue, under conditions of stress and during healing they can alter their metabolism significantly, leading to the secretion of various growth factors [3].

One of the key factors involved is the basic fibroblast growth factor-2 (FGF-2). This multifunctional, 146 amino acid polypeptide has been shown to affect the proliferation, chemotaxis, and differentiation of mesoderm-derived cells [6, 7]. Recent studies have identified it as a potential stimulator of angiogenesis, and this protein has been intensively studied in the wound healing processes of various animals [8–10]. Moreover, other proteins involved in the regulation of angiogenesis, such as vascular endothelial growth factor-A (VEGF-A), have been shown to play important roles in the remodeling of tendons and in their degenerative diseases [11]. Furthermore, VEGF-A has also been shown to stimulate endothelial cell proliferation and permeability [12]. Both of these factors may influence angiogenesis, the activity of which may be measured indirectly through the expression of CD34 antigen. This cell marker is characteristic of endothelial progenitor cells and of blood vessel endothelial cells [13]. Although the majority of these studies confirmed the impact of both FGF-2 and VEGF-A on tendon wound healing, only limited information is available concerning their significance in massaged rat tendons.

In view of the increasingly wide interest in active recreation (including running, biking, and aerobic exercise), which in some situations is associated with excessive overload, trauma of anatomic structures of the locomotor system (muscles, tendons, and ligaments) is diagnosed frequently. A similar tendency can be seen in professional sports [14]. This trauma is probably reflected in the transient insufficiency of the adaptive processes of muscle and tendon tissues [15]. Current knowledge indicates that long-term physical exercise with a high load is followed by a transient decrease in the number and diameter of tendon collagen fibers [16]. This decrease is particularly intense in the period between the third and fifth weeks of training. In subsequent periods, the tendon structure undergoes a transformation leading to an increase in the number and diameter of collagen fibers. This may point to the occurrence of favorable adaptive processes [1, 16]. Such variability in tendon structure during its remodeling may be linked to a significant risk of trauma manifestation during prolonged physical training, especially between the third and fifth weeks of training. In parallel, the studies that have been conducted using ultrastructural morphometry show that multiple mechanical deformations (massage), combined with compression exerted across the long axis of the tendon, are followed by an increase in the number and in the cross-sectional area of collagen fibers in the tendon [17]. This allows the suggestion that prolonged tendon massage can lead to an intensified transformation of tendon tissue, producing an improved trophic situation within the massaged tissue. This may be the result of intensified angiogenesis and the increased metabolic activity of the fibroblasts, which are the main cells responsible for adaptive processes in tendon tissue. In view of the above it would be interesting to resolve the problem of whether massage performed before prolonged physical training or during its course can lead to an increase in fibroblast activity and angiogenesis. This could hypothetically prevent the transient decrease in adaptive capacity processes in the tendon. Recently, it was shown that instrument-assisted cross-fiber massage can increase tissue perfusion and remodel the microvasculature of healing knee ligaments [18]. Although the general opinion is that massage represents a favorable action in the process of preparing the body to the intense physical effort; this suggestion has not yet been fully supported by scientific results. Moreover, no studies have been performed regarding the beneficial effects exerted by tendon massage on angiogenesis processes within this tissue. Therefore, it seems to be important to examine the effect of long-term massage of tendons on their vascularization, which might promote an increase in the number and thickness of the collagen fibers.

Based on this, our study was aimed at resolving the problem of whether tendon massage, applied before or during intense physical training, could stimulate angiogenesis.

## 2. Material and Methods

### 2.1. Animals and Experimental Design

The experiment was conducted at the Animal Research section of the Department of Pathomorphology, Medical University of Wroclaw, Poland. During its course, all the animals were housed in identical conditions. The study was conducted on 75 Buffalo strain rats, all ten months old, randomly divided into three groups of 25 rats each. In the first group (PM, the premassaged group), massage was performed five times a week for 3 weeks before the running training. In the second group (M, the massaged group), massage was applied five times per week for the whole training experiment. The third group (C, the control group) was subjected to running for 10 weeks without massage at all. Rats of all three groups were subjected to running training on an Exer-3R running track (Columbus Instruments, USA) five days per week for ten weeks. The daily duration of training began at 10 minutes on the first day and was increased by 5 minutes every day, until it reached 30 minutes at the end of the first week. Running was carried out at a speed of 0.3 m/s in the first week, and followed by an increased speed of 0.5 m/s in the remaining period of the training. Two groups of rats were subjected to massage. In order to standardize the procedure; the massage was conducted using an algometer head (Digital Algometer Pain Diagnostic Gage, Wagner Instruments, Greenwich, USA) of 0.5 cm^2^ in area, with a constant compression power of 9.81 N (1 kG), using spiral movements along the tendon of the long flexor muscle of the fingers, at the plantar surface of 1 cm^2^ (on each of the rear extremities) [17]. The duration of the massage was 5 minutes per rear leg in each rat.

### 2.2. Euthanasia and Sample Collection

The studies were approved by Local Ethical Commission for the Animal Experiments number 1 in Wroclaw (decision number 5/2011). All possible steps were taken to avoid animal suffering at each stage of the experiment. Rats of all three groups were subjected to anesthesia using ketamine (10 mg/kg body weight) and then sacrificed by decapitation. Tissue material for the studies was sampled from 5 consecutive rats from each experimental group on days 7, 21, 35, 49, and 70 of the experiment. Tissue samples were collected from the middle part of the tendon of the flexor digitorum longus in each rear leg. Each resected tendon was divided into three parts: (1) the first was fixed in 10% buffered formalin, dehydrated, and embedded in paraffin, the second was stored in RNAlater (Qiagen, Hilden, Germany), and the third was frozen in liquid nitrogen and stored at −80°C.

Paraffin sections were stained with haematoxylin/eosin (H&E) and assessed by a pathologist under a BX41 light microscope (Olympus, Tokyo, Japan). The fragments stored in RNAlater and fresh-frozen were utilized for molecular studies.

### 2.3. Immunohistochemistry (IHC)

Immunohistochemical reactions were performed on 4 *μ*m thick tendon sections in an automated staining platform Autostainer Link48 (Dako, Glostrup, Denmark) to ensure constant reaction conditions. In order to deparaffinise, rehydrate, and retrieve the antigens, the sections were boiled in Target Retrieval Solution High pH (9.0) buffer (Dako) using Pre-Treament Link Platform (Dako). Tendon sections were then washed in TBS/0.05% Tween buffer followed by a 5 min incubation with EnVision FLEX Peroxidase-Blocking Reagent to block the activity of endogenous peroxidase. The sections were then subsequently rinsed in TBS/0.05% Tween buffer and incubated with primary antibodies (20 min at room temperature; RT) directed against CD34 (goat anti-rat, R&D Systems, Abington, UK) and VEGF-A (rabbit anti-rat, Antibodies-Online GmBH, Aachen, Germany). Sections were then washed in TBS/0.05% Tween followed by incubation (20 min, RT) with EnVision FLEX/horseradish peroxidase- (HRP-) conjugated secondary antibodies (Dako). In the next step the substrate for peroxidase, diaminobenzidine (Dako) was applied and the sections were incubated for 10 min at RT. Finally, the sections were counterstained with Mayer's haematoxylin, dehydrated in alcohol (70%, 96%, and 99.8%) and xylene, and then mounted using SUB-X Mounting Medium (Dako).

### 2.4. RNA Extraction, cDNA Synthesis, and Real-Time PCR

Total RNA was isolated from 75 tendon fragments stored in RNAlater using RNeasy Fibrous Mini Kit (Qiagen) in line with manufacturers' recommended procedures. Reverse transcription reactions were processed using a High-Capacity cDNA Reverse Transcription Kit (Applied Biosystems, Carlsbad, CA, USA). The relative mRNA expression of* VEGF-A*,* CD34*, and* FGF-2* was evaluated by real-time PCR using the 7500 Real-Time PCR System, primers, and probes of the TaqMan system (all from Life Technologies, Carlsbad, CA, USA). The primers and probes used in the reactions included Rn01511601_m1 for* VEGF-A*, Rn03416140_m1 for* CD34*, Rn00570809_m1 for* FGF-2* and Rn99999916_s1 for* GAPDH*. Real-time PCR reactions were performed on 96-well plates (Life Technologies) at a set time and temperature, including polymerase activation at 50°C for 2 minutes, preliminary denaturation at 94°C for 10 minutes, denaturation at 94°C for 15 s, and annealing of primers, probes, and synthesis at 60°C for 1 min at 40 cycles. The results were standardized based on expression of* GAPDH* and the relative expression (RQ) of the studied genes was calculated using the ΔΔCt method [19]. The sample characterized by the lowest ΔCt was used as a calibrator in all the calculations.

### 2.5. SDS Page and Western Blot

The frozen tissue samples of 75 Buffalo strain rats tendons were homogenized in RIPA lysis buffer (50 mM Tris-Cl pH 8.0, 150 mM NaCl, 0.1% SDS, and 1% Igepal CA-630), 0.5% sodium deoxycholate, a cocktail of protease inhibitors, and 0.5 mM PMSF (all from Sigma, St. Louis, MI, USA). Protein concentration was measured using the BCA technique (Thermo Scientific, Waltham, MA, USA) and a NanoDrop 1000 spectrophotometer (Thermo Scientific). Tissue extracts were mixed with sample buffer (250 mM TRIS pH 6.8, 40% glycerol, 20% (v/v) *β*-mercaptoethanol, 100 mM DTT, 0.33 mg/mL bromophenol blue, and 8% SDS) and denaturated for 10 min at 95°C. Equal amounts of protein (20 *μ*g per lane) were separated by electrophoresis, following Laemmli, in a 12% gel, using the Mini Protean 3 apparatus (BioRad, Hercules, CA, USA). Subsequently, the proteins were electrophoretically transferred to a PVDF membrane (Immobilon P, Millipore, Billerica, MA, USA) and nonspecific binding sites were blocked using 3% BSA in TBST buffer. The amount of the applied protein was controlled by staining the total protein on the membrane using Ponceau S (Sigma). The expression of VEGF-A was detected using specific monoclonal anti-VEGF-A antibody (Dako). Incubation was conducted for 18 h at 4°C with gentle shaking in the solution of the antibody diluted 1 : 200 in 0.3% BSA, in 0.2% TBST (TBS and 20% Tween). After incubation the membrane was washed three times with 0.2% TBST buffer and incubated for 1 h with goat antimouse antibody conjugated with horseradish peroxidase (HRP) (1 : 2000, Jackson Immunoresearch, West Grove, PA, USA). The detection was conducted using a chemiluminescent substrate (LuminataTM Crescendo Western HRP Substrate; Millipore), and the results documented for exposure times ranging from 2 s to 30 min in a Chemi-Doc XRS Molecular Imager apparatus (Bio-Rad). The resulting bands were estimated by densitometric quantitative analysis of protein and normalized to GAPDH levels.

### 2.6. Statistical Analysis

Statistical analysis was performed using Prism 5.0 (GraphPad, La Jolla, CA, USA). The differences between the groups were tested using two-way ANOVA analysis of variance with the Bonferroni multiple-comparison test. Correlations between the examined markers were analyzed using Spearman's correlation test. In all analyses, the results were considered statistically significant when *P* < 0.05.

## 3. Results

### 3.1. Histological Findings

**Figure 1:**
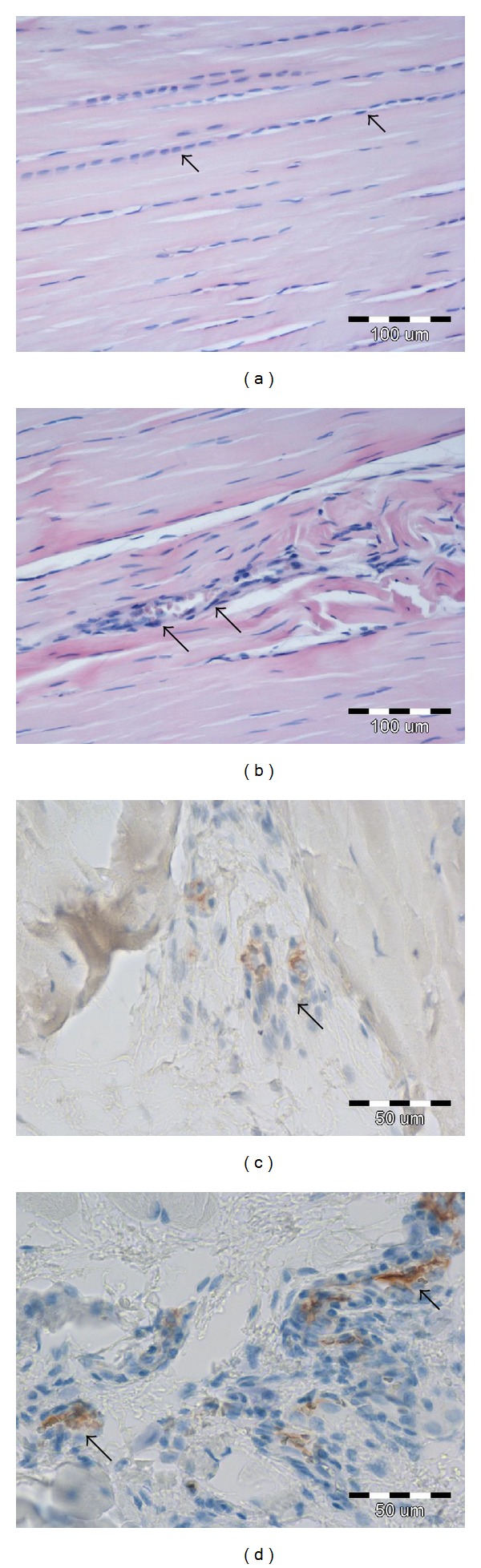
H&E staining. A microphotograph of a rat tendon presenting nuclei of tendon cells (arrows) between parallel plates of collagen fibers. Note: no tissue lesions or inflammatory cells are visible in any of the sampled tendons, regardless of the duration or the experimental group (a). Blood vessels with erythrocytes in the lumen (indicated by arrows) are present in the resected rat tendons (b). IHC staining. In the peritendinous tissues VEGF-A expression was observed in fibroblastic-like cells (indicated by arrow) (c). CD34 expression was noted in blood vessel endothelium (indicated by arrow) (d).

Histological examination revealed no changes of note in the structure of the analyzed tendon, regardless of the duration of the experiment or the study group. No areas of necrosis, calcification, or signs of collagen fiber degeneration or acute inflammation could be seen in any of the analyzed groups (Figures 1(a) and 1(b)). Furthermore, no mitotic figures in the analyzed tendons were noted. Analysis of IHC sections allowed the demonstration of VEGF-A immunoreactivity in the fibroblastic-like cells of peritendinous tissues (Figure 1(c)). In addition, CD34 expression was noted in blood vessel endothelium of the same sections (Figure 1(d)). Due to the low amounts of remaining tissue material for the IHC analysis, no reliable statistical analysis could be performed to compare the expression levels of these markers between the analyzed groups.

### 3.2. Analysis of VEGF-A, CD34, and FGF-2 mRNA Expression

**Figure 2:**
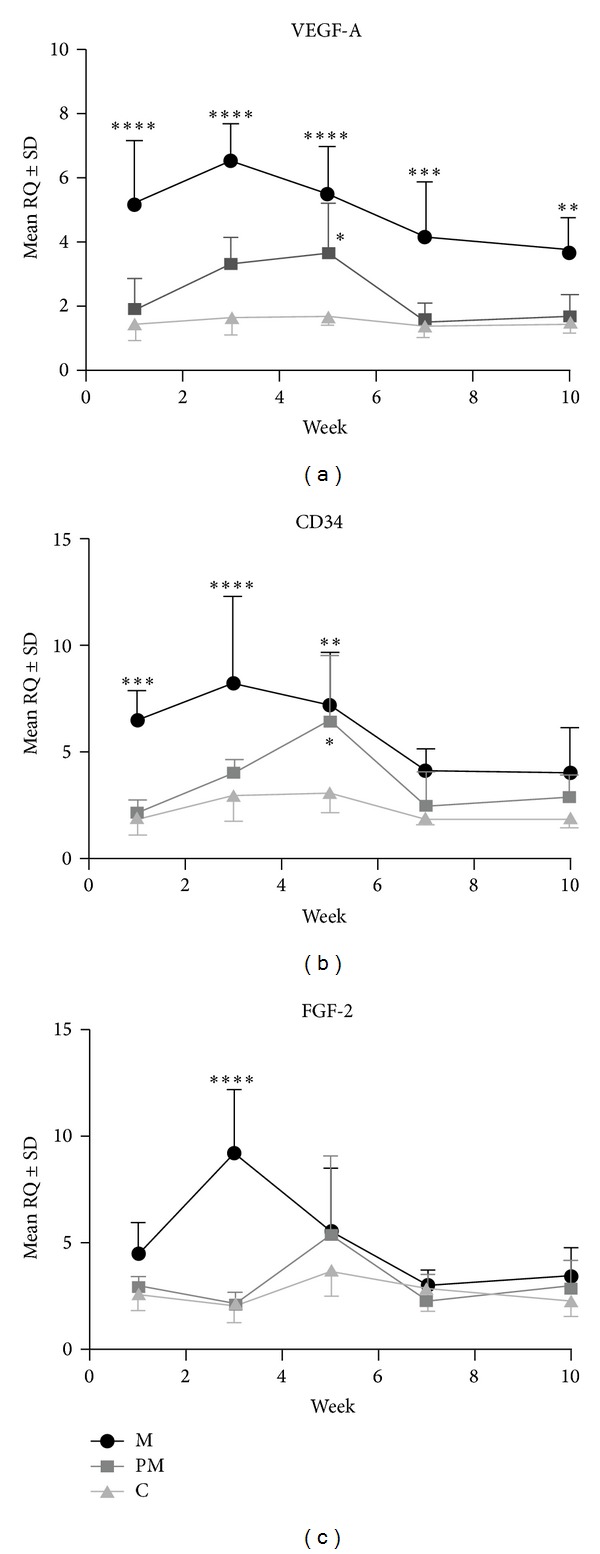
Differentiated mRNA expression of* VEGF-A* (a),* CD34* (b), and* FGF-2* (c) in the massage (M), premassage (PM), and control (C) groups. Significant differences were noted between the M and C and between the PM and C groups. _ _*P < 0.05, _ _**P < 0.005, _ _***P < 0.0005, and _ _****P < 0.0001; Bonferroni multiple comparison test.

Relative expression of the analyzed genes varied significantly with the experiment time and between particular study groups (Figure 2). The highest level of expression of* VEGF-A* mRNA was noted in the massaged group (M) and was significantly higher in relation to the control group (C) in all weeks of the study (*P* < 0.0001 for the first to fifth week, *P* < 0.0005 for the seventh week, and *P* < 0.005 for the tenth week). Similarly, the tendons of the animals in the M group also had significantly higher* VEGF-A* mRNA expression in comparison to those of the premassaged group (PM). Significant differences were noted for the first (*P* < 0.0001), third (*P* < 0.0001), seventh (*P* < 0.0005), and tenth (*P* < 0.05) weeks of the experiment. In the PM group, the* VEGF-A* mRNA expression was significantly higher, as compared to the C group, only in the fifth week of the experiment (*P* < 0.05) (Figure 2(a)). Differences were also noted in* CD34* mRNA expression, the levels of which were highest in the M group, with significant increases in expression in the first (*P* < 0.0005), third (*P* < 0.0001), and fifth (*P* < 0.005) weeks of the experiment, as compared to the C group, and in the first (*P* < 0.0005) and third (*P* < 0.005) week, in comparison to the PM group. The mRNA expression of* CD34* was significantly higher in the PM tendons, compared to the C group, in the fifth week of the studies (*P* < 0.05) (Figure 2(b)). The least pronounced differences were noted in the expression of* FGF-2* mRNA, which was significantly higher in the M group in the third week of the study, as compared to the C and PM groups (both *P* < 0.0001) (Figure 2(c)). Taking the results together, the highest expression of all the analyzed genes in the M group developed in the third week of the study.

### 3.3. VEGF-A Protein Expression

**Figure 3:**
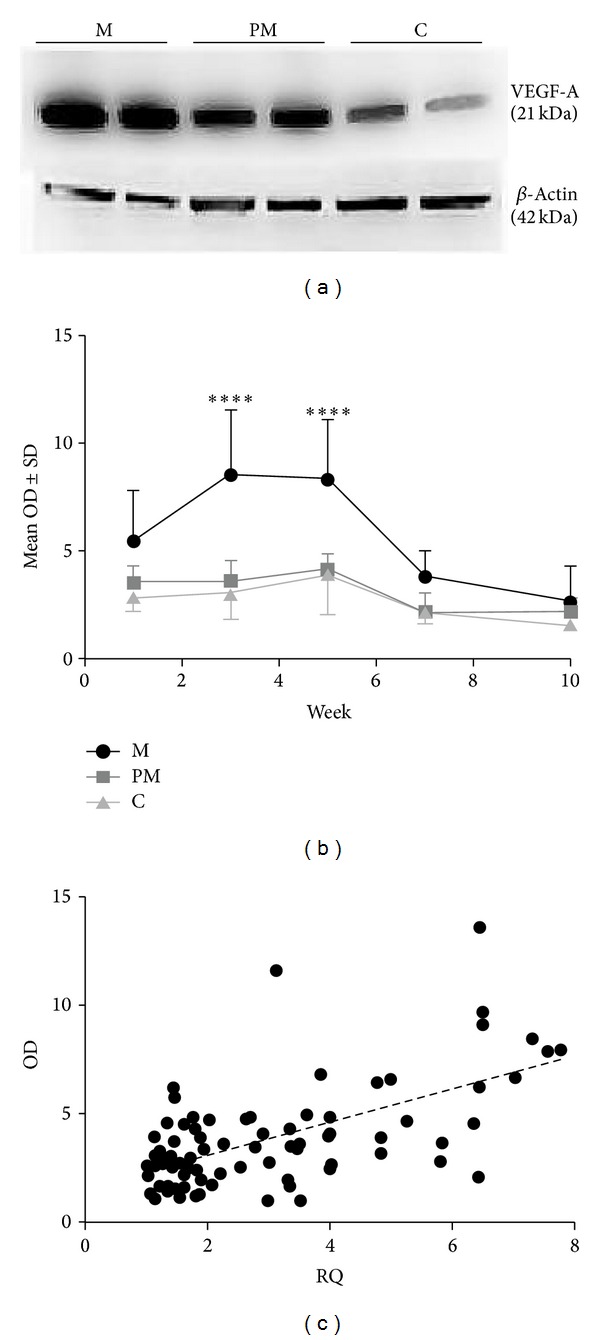
Bands corresponding to VEGF-A 21kDA isoform obtained from tendons sampled in the fifth week of the experiment from the massage (M), premassage (PM), and control (C) groups (a). Optical density (OD) of the 21 kDa VEGF-A protein expression in individual study groups. Significant differences were detected between the M and C study groups. _ _****P < 0.0001; Bonferroni multiple comparison test (b). Correlation between* VEGF-A* mRNA (RQ: relative expression) and protein (OD) expression (*r* = 0.53; *P* < 0.0001; Spearman correlation test) (c).

As the most pronounced differences between the groups were detected in* VEGF-A* mRNA expression, it was decided to confirm our observations on the level of proteins. Using the anti-VEGF-A antibody, we visualized the monomeric form of VEGF-A (21 kDa) (Figure 3(a)). The 21 kDa VEGF-A monomeric form expression was significantly higher in the M group in the third and fifth weeks, than in the C (in both cases *P* < 0.0001) and PM groups (*P* < 0.0001 and *P* < 0.0005, resp.) (Figure 3(b)). The expression of* VEGF-A* mRNA correlated strongly with the optical density (OD) of the obtained bands (*r* = 0.53, *P* < 0.0001, Spearman's correlation test) when the results were pooled from all the weeks of the study (Figure 3(c)). Moreover, as with* VEGF-A* mRNA expression, the highest VEGF-A protein expression was observed in the M group in the third week of the experiment.

## 4. Discussion

It has been shown that physical training can induce structural and functional alterations, not only in the skeletal muscles, but also in tendons [1, 16, 20–26]. These alterations reflect metabolic changes on a subcellular level in tendon tissue, such as increase in collagen synthesis [27–29]. Mechanical stimulation of the tendon during massage is thought to activate fibroblasts, which, through the increase in collagen synthesis, may promote tendon repair following trauma [30, 31]. Therefore, it can be argued that tendons' reactions to mechanical stimuli include favorable alterations in their mechanical properties [32, 33]. Recently, Loghmani and Warden showed that instrument-assisted cross-fiber massage increases tissue perfusion and alters microvascular morphology during regeneration of injured knee ligaments [18]. So far, no data has been available concerning the effects of mechanical stimulation on angiogenesis processes in healthy tendons, which in turn may be of a key importance in the adaptive and regenerative processes of tendons.

Our results demonstrate the most pronounced increases in the expression of* VEGF-A*,* CD34*, and* FGF-2* genes and of VEGF-A protein, in the group of rats subjected to massage (group M) in the third week of the experiment. A slight increase in the expression of* VEGF-A* and* CD34* in the group subjected to massage only prior to the training (group PM) was detected in the fifth week of the experiment. On the other hand, no significant alterations in the expression of the studied factors were seen in the group of rats that was subjected to the training alone, with no preceding or simultaneous massage (group C). This may point to significant adaptive processes in the group of animals that has been subjected to mechanical stimulation (massage) of tendons under load during the running training.

Multiple lines of evidence suggest that tendon fibroblasts subjected to cyclic stretching secrete higher amounts of TGF-*β* (transforming growth factor beta), PGE_2_ (prostaglandin E_2_), and LTB_4_ (leukotriene B_4_), resulting in increases in collagen type I levels and fibronectin production [3]. It can thus be assumed that elements of tendon tissue react to the nonspecific mechanical stimulus (massage) by activating the expression of several mediators, including* FGF-2*, which may initiate changes in the structure and mechanical properties of the tendon [34–36]. In several studies, FGF-2 was shown to stimulate the metabolic activity of fibroblasts, which are the main types of cells in a tendon and are responsible for the synthesis of extracellular matrix proteins. Therefore, the increased expression of FGF-2 observed in this study as result of massage may point to the activation of tendon metabolism and the development of adaptive processes [37–39]. It seems that these adaptive processes may not be ascribed to the proliferation of fibroblasts, as no differences between analyzed groups upon histological examination were noted. However, we have recently shown that the massage of rat tendons modifies their structure by increasing the number of fibers with the smallest diameter (<100 nm), a fact which may indirectly corroborate the results obtained in this study [17].

Although a vast literature is available dealing with the transformation of mechanical stimuli acting on tissues into reactions at the cellular or molecular level [40–43], none of it explains the mechanism by which massage could stimulate angiogenesis in tendon tissue. Interestingly, in our study we observed an increased expression of* VEGF-A* and* FGF-2* in the group of rats subjected to massage. VEGF-A is known to represent one of the most effective angiogenesis-stimulating agents in various types of tissues [12]. FGF-2, in turn, stimulates the metabolic activity of fibroblasts (the main cells in tendons, responsible for the synthesis of extracellular matrix proteins) and has also been shown to be a potent inducer of angiogenesis [8–10, 37–39]. The increase in the expression of both of these markers may explain the observed increase in the expression of* CD34*, a marker of endothelial progenitor cells and endothelial cells, which may indicate the development of new blood vessels, which could be visualized using the IHC method in this study [13]. Moreover, our results may partially explain the results obtained by Loghmani and Warden [18], which showed that the massage of knee ligaments resulted in increased tissue perfusion and alteration in the microvascularity. The highest increase in mRNA expression of all the analyzed markers was observed in case of* VEGF-A*. These changes were also confirmed on the protein level. Therefore it seems that massage may strongly stimulate the expression of these proteins, in addition to* FGF-2*, which in turn may strongly contribute to the adaptive processes of the tendon.

However, it should be stressed that animals utilized in these study were not perfused before sample collection. It is possible, that the observed increase in VEGF-A expression in tendons subjected to massage may be the result of increased number of myeloid-derived cells, for example, monocytes present in the blood vessels. FGF-2 was shown to potentiate leukocyte recruitment in a rat model of acute skin inflammation [44]. Similar effect may be hypothetically caused by massage via increase of its expression. FGF-2 recruited monocytes to the tendon tissues could stimulate angiogenesis, as in inflamed and hypoxic tissues in response to several cytokines [45, 46]. However, further research on the role of myeloid-derived cells in angiogenesis procesess in the tendon is required to elucidate such hypothesis.

It should be noted that the deformation of the tendons during massage was executed with compression across the long axis of the tendon and was thus nonspecific, compared to the deformation developing during distension of the tendon following muscle contraction in the course of the physical activity. It is probably just for this reason that the observed alterations developed in the rats of the group subjected to massage, representing a reaction to the untypical factor acting on the tendon. On the other hand, the transient character of the alterations observed in the expression of* VEGF-A*,* CD34*, and* FGF-2* (which reached their highest values in the third week of the experiment, to be followed by a gradual decrease until the end of the experiment) most probably reflect adaptive processes. This was particularly significant when compared to the unfavorable structural alterations noted in the tendons during the running training and involving a transient (developing between third and fifth weeks of training) decrease in the number, diameter, and cross-sectional area of the tendon-forming collagen fibers [16]. On this basis, the increase in angiogenesis and fibroblast activity induced by mechanical stimulation (massage) of a tendon may be expected to prevent the transient tendency for its structure to weaken. This may decrease the development of lesions within tendons during their long-term overload in the course of running.

## 5. Conclusions

The obtained results may point indirectly to a beneficial effect of massage conducted during long-term intense physical effort on tissue metabolism in the tendon. Moreover, the increased metabolism in the tendons may exert a protective effect in situations where there is an increased risk of tendon damage from unfavorable structural alterations developing during long-term high-intensity physical effort, such as running. However, further studies on the molecular and structural levels are required to fully clarify the mechanisms behind the changes developing in tendon tissue subjected to massage in the course of strenuous physical effort.
